# Influence of Sulfurization Time on Sb_2_S_3_ Synthesis Using a New Graphite Box Design

**DOI:** 10.3390/ma17071656

**Published:** 2024-04-04

**Authors:** Sheyda Uc-Canché, Eduardo Camacho-Espinosa, Ricardo Mis-Fernández, Mariely Loeza-Poot, Francisco Ceh-Cih, Juan Luis Peña

**Affiliations:** Centro de Investigación y de Estudios Avanzados del IPN, Unidad Mérida, Departamento de Física Aplicada, Km. 6 Antigua Carretera a Progreso, Mérida 97310, Yucatán, Mexico; eduardo.camacho@cinvestav.mx (E.C.-E.); mariely.lopt@gmail.com (M.L.-P.); francisco.ceh@cinvestav.mx (F.C.-C.); jlpenachapa@gmail.com (J.L.P.)

**Keywords:** Sb_2_S_3_ absorber, sulfurization, two-step process, graphite box, annealing, p-n junction

## Abstract

In recent years, antimony sulfide (Sb_2_S_3_) has been investigated as a photovoltaic absorber material due to its suitable absorber coefficient, direct band gap, extinction coefficient, earth-abundant, and environmentally friendly constituents. Therefore, this work proposes Sb_2_S_3_ film preparation by an effective two-step process using a new graphite box design and sulfur distribution, which has a high repeatability level and can be scalable. First, an Sb thin film was deposited using the RF-Sputtering technique, and after that, the samples were annealed with elemental sulfur into a graphite box, varying the sulfurization time from 20 to 50 min. The structural, optical, morphological, and chemical characteristics of the resulting thin films were analyzed. Results reveal the method’s effectivity and the best properties were obtained for the sample sulfurized during 40 min. This Sb_2_S_3_ thin film presents an orthorhombic crystalline structure, elongated grains, a band gap of 1.69 eV, a crystallite size of 15.25 Å, and a nearly stoichiometric composition. In addition, the formation of a *p-n junction* was achieved by depositing silver back contact on the Glass/FTO/CdS/Sb_2_S_3_ structure. Therefore, the graphite box design has been demonstrated to be functional to obtain Sb_2_S_3_ by a two-step process.

## 1. Introduction

Chalcogenide materials such as Sb_2_S_3_ have attracted considerable attention because of their outstanding properties as a photovoltaic absorber layer. This material has a direct band gap of around 1.7 eV and an extinction coefficient of 1.8 × 10^5^ cm^−1^ [1], making it suitable for solar cell fabrication. Furthermore, Sb_2_S_3_ is a stable material composed of earth-abundant and environmentally friendly elements [2,3].

Sb_2_S_3_ thin films are typically obtained using a one-step process using different deposition methods, among them sputtering, chemical bath deposition (CBD) [4], thermal evaporation (TE), rapid thermal evaporation (RTE), and close space sublimation (CSS) [5,6]. In these techniques, crystalline structures with uniform and thicker thicknesses are obtained. However, they produce non-stoichiometric thin films compared to the source material, resulting in Sb-rich thin film and leading to defect formation (sulfur vacancies) [7,8]. Being the main cause of this decompensation the Sb_2_S_3_ high vapor. To compensate for the sulfur pressure loss, an additional annealing in a sulfur atmosphere (sulfurization) is required [8,9,10].

An alternative approach to Sb_2_S_3_ synthesis is a direct two-step process, beginning with a metallic Sb source deposited using a vacuum method, followed by a thermal treatment in a sulfur atmosphere [11]. This fabrication process is cost-effective, easy to implement, and offers the advantage of sulfur loss compensation and vacancy reduction [12]. The previous work that implemented a two-step process includes the one presented by Lei et al. [13], who deposited metallic Sb by RF-sputtering with a subsequent in situ hydrothermal process to obtain Sb_2_S_3_. Additionally, L. Zhang et al. deposited metallic Sb thin films using the TE method and performed annealing in nitrogen/hydrogen sulfide (N_2_/H_2_S) atmosphere to form Sb_2_S_3_ [12]; while J. Zhang et al. obtained Sb_2_S_3_ by low-temperature plasma sulfurizing metallic Sb using sulfur powder varying the radiofrequency power conditions [14].

Furthermore, there are other chalcogenide materials that require sulfurization using a two-step process [15,16]. In these cases, successful sulfurization has been achieved using a graphite box in the presence of sulfur powders. Regularly, in conventional approaches, graphite boxes are typically designed with square or circular geometries and feature holes along the edges close to the sample where the sulfur powder or pellet precursors are placed. These designs lead to sulfurización with the interaction between the sublimated sulfur and the metallic precursor using a convective flux that serves as carrier gas. It should be noted that this process has been scarcely explored in the fabrication of Sb_2_S_3_ thin films. One advantage of employing sulfur powder as a source and graphite box is that it minimizes the risk of poisoning compared with other more toxic sources (H_2_S gas) [17].

Experimental conditions that significantly affect the Sb_2_S_3_ thin film properties using a two-step process, such as time, pressure, sulfur content, and the thin metallic film thickness, should be taken into consideration to improve the optoelectronic properties [18]. Particularly, in this work, Sb_2_S_3_ thin films were obtained using a two-step process within a new graphite box design and varying the sulfurization time. The new graphite box design strategically places the antimony sample directly above a layer of sulfur powder, enabling more effective and direct sulfurization via convective interaction. This design ensures that the sublimated sulfur has a shorter path to interact with the antimony sample and could probably generate a better Sb_2_S_3_ material. The graphite box design allows direct interaction of the sulfur with the sample, which could decrease certain sulfurization parameters, such as temperature, time, and sulfur precursor content. The main disadvantage of this process could be the total sulfurization of metallic Sb.

The structural, optical, morphological, and chemical changes were studied. To validate the efficacy of this new method, we have also demonstrated the formation of a *p-n junction* using CdS and the synthesized Sb_2_S_3_. While it is acknowledged that the solar cell efficiency of the resulting device is not optimized, it serves only as a proof of concept. The primary contribution of this study remains in the innovative approach to sulfurization, which opens new possibilities for the accurate and efficient production of Sb_2_S_3_ and to obtain other chalcogenides.

## 2. Materials and Methods

**Figure 1:**
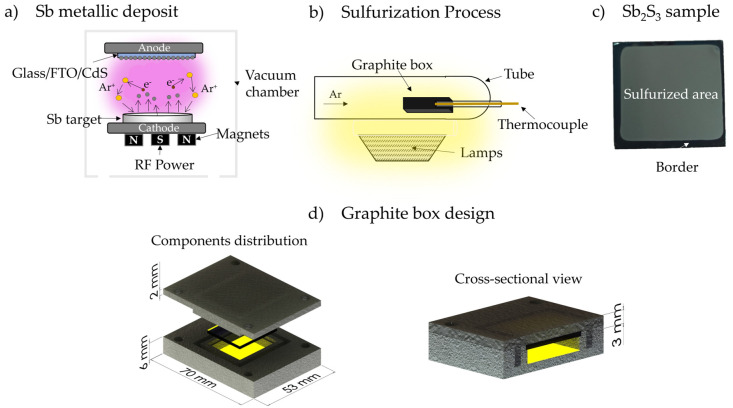
(**a**) Schematic illustration of Sb_2_S_3_ fabrication process using a two-step process: as-deposited and (**b**) its subsequent sulfurization, (**c**) Sb_2_S_3_ sample, and (**d**) graphite box design.

Sb_2_S_3_ samples were fabricated on a 1 × 1 in^2^ Glass/FTO/CdS structure. The cleaning process began with washing fluorine-doped tin oxide glass substrate (FTO-TEC15-Sigma Aldrich, St. Louis, MI, USA) using soap and water; then, they were cleaned by rinsing in ultrasonic baths of acetone and methanol, followed by N_2_ drying. After the cleaning, CdS and Sb deposition by RF-Sputtering at room temperature. Firstly, CdS thin films were deposited using a commercial 4N-purity CdS target, with the deposition parameters set at a sputtering power of 90 W Ar pressure of 25 mTorr for 17 min, resulting in a final thickness of 120 nm. Subsequently, antimony thin films with an approximate thickness of 450–500 nm were obtained using a commercial 4N-purity Sb target. From this point, the two-step process is considered to begin (Figure 1a). The Glass/FTO/CdS structures were placed into a vacuum chamber at a fixed distance of 10 cm from the target. The chamber was evacuated, achieving a base pressure of 1 × 10^−5^ Torr. The deposition was performed at an Ar pressure of 10 mTorr and a power of 30 W for 20 min. Finally, (Figure 1b), the structures were sulfurized using 60 mg of elemental sulfur powder (reactive sulfur with 99.5% purity content), which was placed and uniformly distributed into a centering rectangular groove in the graphite box bottom. Moreover, the box has flanges where the sample is placed so that the Sb thin film side is positioned directly above the sulfur powder. The box is closed with a graphite lid that is adjusted with graphite screws; a schematic representation of the innovative graphite box design is illustrated in Figure 1c.

During the sulfurization process, the graphite box is placed into a furnace tube under 650 Torr of Ar atmosphere and heated at a rate of 20 °C/min until reaching 300 °C. Sulfurization times were 20, 30, 40, and 50 min. After sulfurization treatment, the sample surface suffered visible changes; at the edges, there is a darker coloration than in the center (Figure 1d). This difference between the edge and the sulfurized area is a result of the graphite box design, which covers the border, leaving it without sulfurization.

### Measurement and Characterization

The structural, optical, morphological, and chemical changes in Sb_2_S_3_ films were determined as follows. The crystal structure was determined using X-ray diffraction (XRD). The diffraction patterns were obtained using Bragg incidence diffraction in a Siemens D-5000 diffractometer (Munich, Germany); the measurement was performed in 2θ sweeping from 10 to 70 degrees with a scan speed of 0.02 degree/3 s, using a Cu kα tube (λ = 1.5406 Å). The optical characterization was measured to determine the band gap in an experimental setup composed of a Xenon lamp of 100 W, a Newport Oriel Cornerstone monochromator model C8130B-1-MC, and 918D-IR-OD3R photodetectors in a wavelength range from 600 to 1000 nm. A Jeol JSM-7600F Field Emission Scanning Electron Microscope (FE-SEM, Tokyo, Japan) was used to obtain superficial and cross-sectional images, with elemental analysis using Energy Dispersive X-ray Spectroscopy (EDS). The chemical composition properties were analyzed using X-ray Photoelectron Spectroscopy (XPS) measurements in Thermo Scientific (Waltham, MA, USA) K-Alpha equipment with an Al X-ray source, calibrated using the C1s photoemission at 284.6 eV. Finally, the photovoltaic effect was proved in the sample with suitable properties placed Ag paint dots on it (0.007 cm^2^). The J-V curves were measured with a Keithley 2420 source meter under 100 mW/cm^2^ light irradiation generated with a halogen lamp and homemade LabView^TM^ software.

## 3. Results and Discussion

### 3.1. Structural Analysis

**Figure 2:**
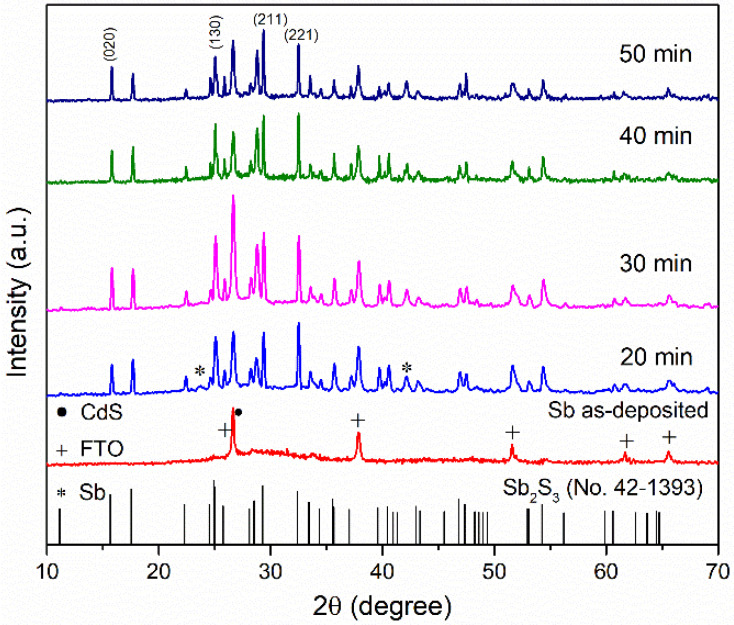
XRD patterns of sample as-deposit and subsequent sulfurization process at different times.

XRD patterns of Sb thin film for as-deposited and after sulfurization at different times can be observed in Figure 2. All samples show peaks corresponding to underlying CdS and FTO layers. The as-deposited sample did not show any peaks associated with Sb, suggesting that an amorphous phase could facilitate the reaction between sulfur and antimony. After sulfurization at different times (20–50 min), the main peaks in (020), (130), (211), and (221) appear, corresponding to orthorhombic Sb_2_S_3_ according to JCPDS card No. 42–139. The method proposed (two-step process and graphite box) in this work is effective for Sb_2_S_3_ formation. On the other hand, after sulfurization at 20 min, it was possible to identify small peaks related to metallic Sb peaks, indicating that the sample has not been wholly sulfurized and probably recrystallized [11,12,13,14]. However, these peaks’ intensity decreases as the time increases to 30 and 40 min, while at 50 min, the Sb peak shows a slight increase in intensity.

The crystallite size has been calculated using the Debye-Scherrer formula [10].

(1) $$D=\frac{k\lambda }{\beta_{hkl}cos\theta }$$

*D* is the average crystallite size, *k* is the factor shape of the crystal, *λ* is the X-ray wavelength (0.15406 nm), *β_hkl_* the full-width half maximum (FWHM), and *θ* is the Bragg’s angle of the X-ray. The crystallite size was calculated from (211) orientation (this orientation was chosen because it is a well-defined and intense peak), and the values are shown in Table 1. The estimated thin film crystallite size increases from 7.51 to 15.25 Å, revealing a direct correlation between crystallite size and sulfurization time. However, after 50 min, the crystallite size decreases, which could be due to the crystallinity reduction. It is possible that the re-evaporated sulfur atoms, due to longer sulfurization time, migrate to the grain boundaries, causing that them to act as barriers limiting the growth of the crystallites [19]. In general, the structural analysis demonstrates polycrystalline Sb_2_S_3_ nature, with 40 min the optimum time for the crystallite’s growth.

### 3.2. Optical Analysis

**Figure 3:**
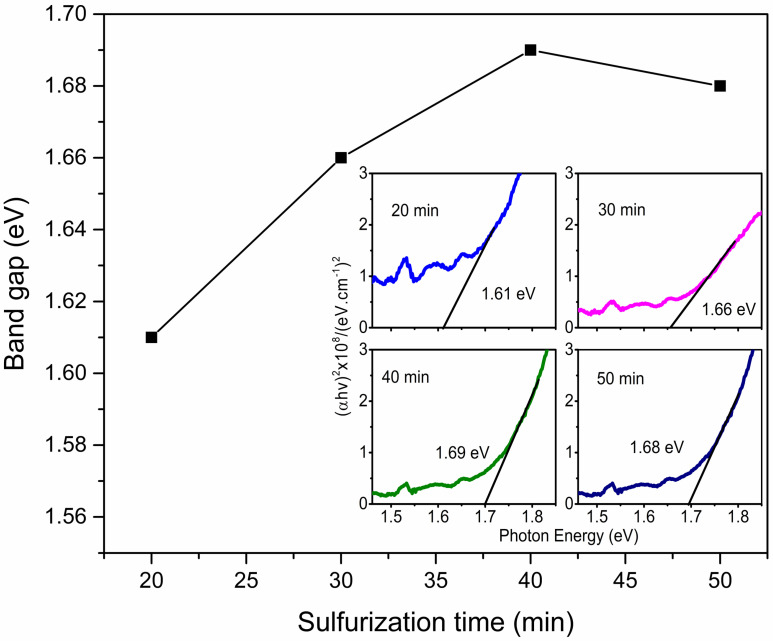
Band gap as a function of sulfurization time and Tauc plot used to determine them (inset image).

The optical characterization has been investigated using a VIS/NIR spectrophotometer. The band gap of Sb_2_S_3_ thin films has been estimated according to the Tauc plot by the next equation [20]:

(2) $$\alpha hv=C{\left(hv-E_{g}\right)}^{n}$$

where α is the absorption coefficient, *h* is Planck’s constant, *ν* is the light frequency, *C* is a constant, and *n* represents the type of electronic transition, being 1/2 or 2 for direct or indirect transition, respectively [7]. Considering *n* = 1/2, the direct band gap of Sb_2_S_3_ samples was determined and is presented in Figure 3. These values are in a range from 1.61 to 1.69 eV (Figure 3 inset), showing a relative increment as sulfurization time rises until 40 min, while at 50 min, a slight decrease is observed. These values are consistent with those reported by other authors [1,10,21,22]. The lowest value found at 20 min could be due to metallic Sb presence, and the different composition, as XRD analysis suggested. The sample sulfurized at 40 min employing the graphite box is close to the characteristic material band gap value, indicating that under these experimental conditions, it is possible to obtain absorber material for photovoltaic applications [1].

### 3.3. Morphological Analysis

**Figure 4:**
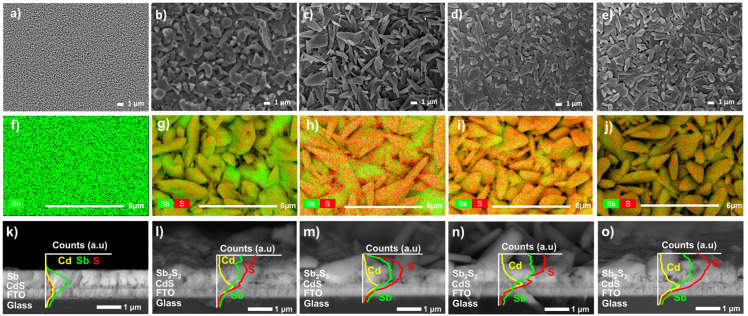
FE-SEM images of surface morphology of sample: (**a**) as-deposited and sulfurization at (**b**) 20 min, (**c**) 30 min, (**d**) 40 min, and (**e**) 50 min. EDS elemental mapping and cross-sectional with EDS line scan of sample: (**f**,**k**) as-deposited and sulfurization of (**g**,**l**) 20 min, (**h**,**m**) 30 min, (**i**,**n**) 40 min, and (**j**,**o**) 50 min.

In the as−deposited sample (Figure 4a), it is possible to appreciate the formation of the small grains (0.15 µm average size); compact and uniform Sb surface, similar morphologies have been found in deposits such as those reported by Lou et al. [23]. Figure 4b–e shows the FE-SEM images of the sulfurized thin films at different times with grain sizes from 1 to 2 µm average. It is possible to observe the formation of elongated-shaped grains associated with Sb_2_S_3_ formation as the sulfurization time increases; as suggested by XRD results, this grain shape has also been observed in TE deposits [24]. On the other hand, the morphology changes from spheric to elongated shapes until 30 min (Figure 4b,c). In the samples at 40 min (Figure 4d), the grains coalesce and present uniform, dense, and flake shapes. At 50 min, irregular shapes and size grains are observed (Figure 4e); consequently, it could affect the material’s electrical properties due to the recombination [25]. Therefore, the 40-minute sulfurized sample shows the best morphological properties.

**Figure 5:**
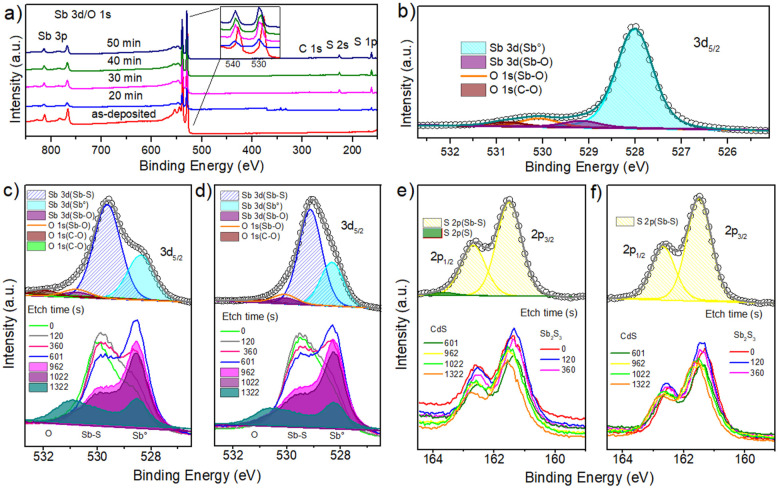
(**a**) XPS survey spectra of samples; (**b**) as-deposited; and the high-resolution and etch time spectra of samples sulfurized at (**c**) 20 min (Sb 3d_5/2_), (**d**) 40 min (Sb 3d_5/2_), (**e**) 20 min (S 2p_3/2_), and (**f**) 40 min (S 2p_3/2_).

The EDS elemental mapping composition of the thin films is presented in Figure 4f–j; the green and red colors are used to show Sb and S, respectively. Figure 5a (as-deposited sample) exhibits only the presence of metallic Sb. The sample sulfurized at 20 min reveals an Sb-rich surface, suggesting a partial S reaction with Sb, as indicated in the 20 min XRD pattern. As the sulfurization time increases to 30 and 40 min, the Sb and S mixture appears relatively homogeneous, which could confirm the expected composition in most of the samples. Finally, the sample sulfurized at 50 min shows Sb predominance, indicating again the presence of small S particles that have not been able to react or have been re-evaporated in the heat treatment process [26].

Figure 4k–o shows the cross-sectional FE-SEM images with their corresponding EDS line scan tracking for the Cd (orange), S (yellow), and Sb (green) samples before and after the sulfurization process. Figure 5f shows that the cross-section film is uniform and compact, completely Sb-composed on Glass/FTO/CdS structure. However, after Sb thin film sulfurization, an important change in grain shape and distribution can be observed (Figure 4i–o). The film thicknesses range from 0.4 to 1.7 μm; such a large thickness variation is a consequence of recrystallization and the Sb_2_S_3_ formation, as J. Zhang suggests [14]. The qualitative analysis using EDS lines confirms the sulfur diffusion through the Sb thin film in all the samples.

Table 2 summarizes the elemental composition and the corresponding S/Sb atomic ratio in a cross-sectional Sb_2_S_3_ area. The 20 min sulfurized sample indicates an Sb-rich composition, suggesting insufficient sulfurization time, and despite the 30 min sample, the ratio increase is still out of the stoichiometric. The 40 min sulfurization time gives an atomic ratio close to 3:2 (S/Sb = 1.5); therefore, using this method and time leads to the Sb2S3 material formation; reaching these stoichiometric conditions probably reduces sulfur vacancies and improves the crystalline quality, as Yang et al., report in their work [15]. On the other hand, the 50-minute sample presents a decrease in the S/Sb ratio; this could be related to S re-evaporated, indicating that the sulfurization time has been excessive. In annealing such as sulfurization, sulfur would be expected to be released when the temperature or treatment time increases, leaving S defect in the films [27].

### 3.4. Chemical Analysis

The chemical changes produced by Sb thin film sulfurization were determined using X-ray photoelectron spectroscopy (XPS). All spectra were calibrated with the C1s peaks at 284.6 eV [28]. The XPS survey spectra of thin films are shown in Figure 5a, and the characteristic peaks of Sb, S, O, and C are indicated in Figure 5b–f. Surveys comparison shows Sb mean peak shift to the higher binding energy upon sulfurization (inset of Figure 5a), probably associated with an Sb_2_S_3_ semiconductor formation. Figure 5b shows the high-resolution at zero time, the peaks of Sb3d and O1s in the Sb precursor, where the Binding Energy (BE) overlap; these regions are 529.2–529.6 eV and 528.1–531 eV, respectively [29]. The BE of Sb3d_5/2_ were found in 528.01 eV and 529.16 eV are associated with the presence of the oxidation states Sb^0^ and Sb^+3^ corresponding to elemental Sb and Sb oxide, respectively [30,31]. Additionally, O1s peaks are identified, associated with the Sb-O bond (530.07 eV) and CO_2_ (530.80 eV). The presence of these two species is due to their interaction with the environment [32]. One way to remove these contaminants is to apply erosion to the samples for XPS measurement or use heat treatments, as reported by other authors [33,34].

Figure 5c,d shows the high-resolution spectra of Sb3d_5/2_, O1s, S2p_1/2_, and S2p_3/2_ with their etch time for the samples sulfurized at 20 and 40 min. Figure 5c shows a peak with a shoulder, split into two prominent peaks, the first at 529.5 eV (Sb3d_5/2_), corresponding to Sb^3+^ in Sb_2_S_3_ [35]. In contrast, the second peak at 528.3 eV (Sb3d_5/2_) indicates the presence of Sb in the elemental state (Sb^0^) because of metallic Sb partial sulfurization [31], as noted in the analyses discussed above. The peak at lower BE observed at 530.7 eV was attributed to Sb^3+^ in Sb-O bonds, mainly Sb_2_O_3_ [30,36]. This observation is confirmed using the deconvolution of the oxygen peak in 531–533.5 eV [36]. The XPS etch time from 0 to 1323 s (Figure 5c,d) was performed to observe the Sb, O, and S distribution change. The Sb_2_S_3_ formation on the bulk is clearly observed. As the etch time increases, the Sb_2_S_3_ peak decreases, and the Sb^0^ peak increases until reaching the maximum point where the Sb^0^ peak is higher (601 s) due to partial sulfurization of the Sb thin film. For etching times greater than 601 s, the interface with the CdS is observed. It is worth highlighting that close to the CdS interface, the Sb_2_S_3_ presence is still observed, probably due to the S diffusion from CdS. Sulfur diffusion can be explained by Fick’s law, which describes how S atoms migrate from a higher to a lower concentration region. This diffusion is due to the random thermal motion of the particles [37]. The motion of atoms through Fick’s law can be described in two ways: the transport of atoms from the solution to the thin film surface or the reaction of the growing species on the surface [4]. Extrapolating the phenomena observed in this study, several authors have reported S diffusion in the absorber layer [38,39].

The high-resolution spectra of S2p (Figure 5e) show a peak that can be deconvoluted into two peaks at 161.4 eV (2p_3/2_) and 162.6 eV (2p_1/2_), associated with S^2−^ oxidation state in Sb_2_S_3_ film [8,40]. Furthermore, a small peak was found at 163.6 eV (S2p_3/2_) in the 20-minute sample (Figure 5e), associated with elemental S, confirming that some sulfur ions did not react with the Sb ions. Figure 5e,f shows the etch time of the S peak distribution from 0 to 1322 s, corresponding to the Sb-S bond. As observed in the Sb peak, an Sb-S bond decrease is also shown at etching time 601 s. However, at 962 s, the S2p doublet shift is evident, suggesting the CdS presence.

The comparison between the sulfurized times at 20 and 40 min shows that the shoulder in the Sb3d peak is more pronounced in the sample treated at 40 min than in the shorter time. This observation is consistent with the EDS analysis, showing better stoichiometry in the sample treated at 40 min. On the other hand, the S2p peak associated with the Sb-S bond did not show other elements for the 30, 40, and 50 min samples. Table 3 summarizes each deconvoluted peak found in the 20 and 40 min samples. In summary, XPS analysis again demonstrates S-Sb bond formation and a partial sulfurization of Sb thin film close to the CdS interface, validating the Sb_2_S_3_ obtention by the method employed in upper layers.

### 3.5. Photovoltaic Effect Measurement

**Figure 6:**
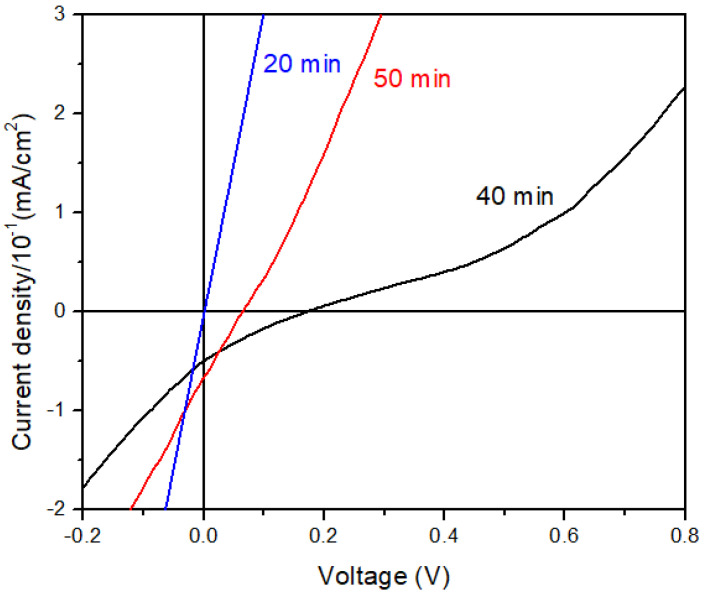
*J-V* Characterization at different sulfurization times.

Structural, optical, morphological, and chemical characterization confirms the Sb_2_S_3_ formation using the proposed two-step process using the designed graphite box. In addition, the new design confirms a decrease in parameters such as pressure, temperature, and sulfur content in the sulfurization process compared to those reported by other authors [15,16,41]. The results indicate that the sample sulfurized at 40 min presents the appropriate properties to be used as a photovoltaic-absorber material. Based on the above, it was decided to corroborate the p-n junction in the obtained sample at 20, 40, and 50 min (Figure 6). The electrical results showed a V_oc_ of 174 mV, highlighting that a p-n junction has been formed to the sample sulfurization at 40 min, while the others have a resistive behavior. Despite having obtained low electrical parameters, it has been reported that fabricating Sb_2_S_3_-based solar cells is challenging, and proof of that is the record of low efficiencies obtained so far [24,42,43,44]. In this case, the photovoltaic parameter deficiency can be attributed to Sb partial sulfurization. Therefore, the metal diffusion through the entire structure causes R_sh_ to diminish, reducing the current and voltage flow through the solar cell junction. Therefore, further optimization of the Sb_2_S_3_ thin film by this method would improve the efficiency of future work.

## 4. Conclusions

Employing the method proposed in this work, including the new graphite box design, allowed for the reduction in some sulfurization parameters. On the other hand, the formation of Sb_2_S_3_ was achieved starting from a Glass/FTO/CdS/Sb structure that was sulfurized at different times. Structural, optical, morphological, and chemical changes were observed with the sulfurization time variation. XRD patterns revealed the Sb_2_S_3_ orthorhombic phase formation. The calculated band gap from optical measurement resulted in values close to 1.7 eV, which are typical for Sb_2_S_3_ material. Both the top surface and the cross-sectional morphology exhibit elongated grains and a thickness increase after sulfurization. XPS analysis confirms a Sb-S bond and Sb^0^, corresponding to Sb_2_S_3_ material formation and metallic Sb, respectively. Being 40 min, the optimum sulfurization time compared with the other times, where the material shows the appropriate properties to be used as absorbent material. The *J-V* characterization was derived at a voltage of 174 mV, proving that it is suitable as an absorber material in a *p-n* junction.

## Figures and Tables

**Table 1:** Crystalline size of (211) orientation.

Sulfurization Time (min)	Crystallite Size (Å)
20	7.51
30	7.58
40	15.25
50	14.78

**Table 2:** S/Sb ratio of sulfurized samples using EDS analysis.

Sulfurization Time (min)	Elements		
	Sb (Atomic %)	S (Atomic %)	S/Sb
20	47.91	52.91	1.10
30	42.62	57.38	1.34
40	41.16	58.84	1.42
50	45.34	54.66	1.20

**Table 3:** XPS binding energy for all peaks fitted of samples sulfurized at different times.

Sulfurization Time (min)	Sb_2_S_3_			Sb_2_O_3_		Sb^0^	Other Elements		
	Sb 3d_5/2_	S 2p_3/2_	S 2p_1/2_	Sb 3d_5/2_	O1s	Sb 3d_5/2_	O1s	O1s	S 2p_3/2_
As-deposited	---	---	---	529.16	530.07	528.01	530.80	---	---
20	529.54	161.49	162.67	530.79	530.78	528.39	531.97	533.21	163.64
30	529.51	161.62	162.80	530.94	530.91	528.48	532.11	---	---
40	529.38	161.48	162.66	530.60	530.63	528.33	531.85	---	---
50	529.44	161.50	162.68	530.81	530.80	528.40	531.97	---	---

## Data Availability

Data will be made available on request.
